# HIS-based electronic documentation can significantly reduce the time from biopsy to final report for prostate tumours and supports quality management as well as clinical research

**DOI:** 10.1186/1472-6947-9-5

**Published:** 2009-01-20

**Authors:** Bernhard Breil, Axel Semjonow, Martin Dugas

**Affiliations:** 1 Department of Medical Informatics and Biomathematics, University of Münster, Domagkstraße 9, 48149 Münster, Germany; 2 Department of Urology, Prostate Centre, University Clinic Münster, Albert-Schweitzer-Straße 33, 48149 Münster, Germany

## Abstract

**Background:**

Timely and accurate information is important to guide the medical treatment process. We developed, implemented and assessed an order-entry system to support documentation of prostate histologies involving urologists, pathologists and physicians in private practice.

**Methods:**

We designed electronic forms for histological prostate biopsy reports in our hospital information system (HIS). These forms are created by urologists and sent electronically to pathologists. Pathological findings are entered into the system and sent back to the urologists. We assessed time from biopsy to final report (TBF) and compared pre-implementation phase (paper-based forms) and post-implementation phase. In addition we analysed completeness of the electronic data.

**Results:**

We compared 87 paper-based with 86 electronic cases. Using electronic forms within the HIS decreases time span from biopsy to final report by more than one day per patient (p < 0.0001). Beyond the optimized workflow we observed a good acceptance because physicians were already familiar with the HIS. The possibility to use these routine data for quality management and research purposes is an additional important advantage of the electronic system.

**Conclusion:**

Electronic documentation can significantly reduce the time from biopsy to final report of prostate biopsy results and generates a reliable basis for quality management and research purposes.

## Background

Assessing advantages and disadvantages of electronic documentation is important to improve hospital information systems. Paper-based documentation in clinical routine is still very common because of the time needed to fill in electronic forms [1] and reluctance to change the documentation routine [2]. On the one hand users are satisfied with the use of routine electronic data [3], on the other hand the subjective opinion whether electronic forms are faster differs [2].

A recent study concerning ambulatory care in the United States shows that although "Electronic health records have the potential to improve the delivery of health care services [...] electronic systems had been adopted by only a small minority of U.S. physicians" and only "four percent of physicians reported having an extensive, fully functional electronic records system, and 13% reported having a basic system" [4]. In inpatient health care there are more electronic systems but the Electronic Health Record (EHR) "must be used by clinicians, and this remains a major challenge" [5].

Moreover user acceptance is strongly influenced by the perceived usefulness of a system [6]. Therefore it is important to assess objective criteria like the turnaround time (TAT), such as time from receipt of a specimen to the availability of a result [7], to evaluate an electronic order-entry system. TAT is a frequently used indicator of efficiency of pathological test ordering [7-10] although the definition of TAT may change. In contrast to laboratories, physicians "perceive TAT as starting at the time the order is written and ending when viewing results" [11]. The second definition is more appropriate in our setting, where we are addressing biopsies of prostate tumours. In addition to pathological findings, a final report is created by the urologist. Therefore we introduced time from biopsy to final report (TBF) as an indicator of effectiveness. Because we analysed the whole process we measured TBF in days and not in minutes.

The aim of this study is to compare TBF between a paper-based and an electronic system. We present an approach in which two paper forms previously used for documentation of prostate biopsies are transferred into the HIS to compare these forms with HIS-based electronic forms.

This system enables shared documentation of physicians from different departments as well as electronic orders of histological examinations for prostate biopsies and therefore constitutes a computerized physician order entry system (CPOE).

There's vast literature concerning CPOE systems whereby most studies describe their usage for medication and prescriptions [12-18] in some cases even combined with clinical decision support (CDS) systems [14,19,20].

Kaushal [21], Khajouei [22] and Wolfstadt [20] reviewed many CPOE studies which are mainly addressing medication and drug applications and focus on medication errors. Many CPOE studies analyse risks for patient safety, patterns of medical orders or effectiveness by TAT but don't measure the full process time from biopsy to the final report [14,17,21,23,24].

Our main goal was to assess a specific order-entry system regarding documentation of prostate histologies involving urologists, pathologists and physicians in private practice. Therefore we concentrated on the following objectives:

1. Is there a measurable time difference regarding TBF between electronic and paper-based documentation?

2. What level of data quality is achieved in routine documentation of prostate biopsies?

3. What benefits of the electronic documentation can increase physicians' acceptance?

## Methods

**Figure 1:**
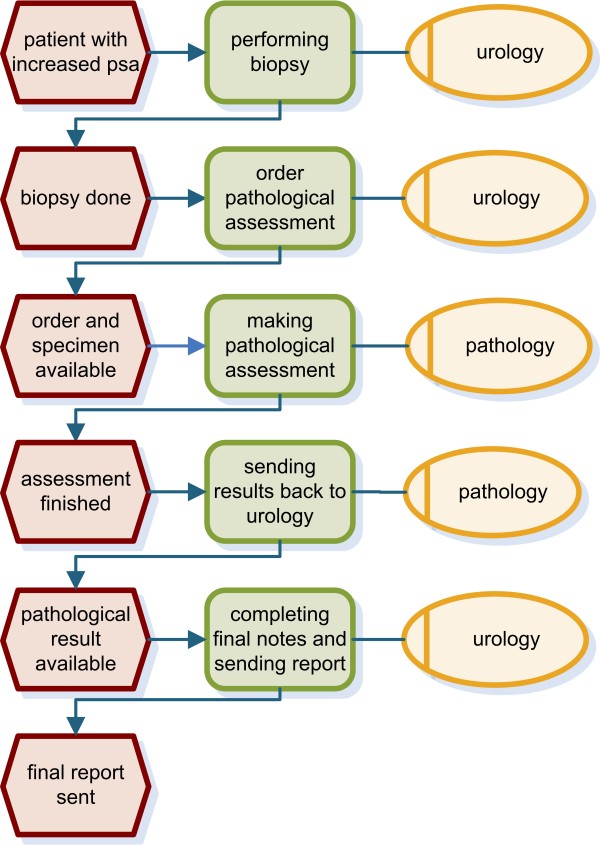
**EPC of the diagnostic process**. An urologist performs the biopsy, which is sent to the pathology department. Pathological findings are combined with urologist's recommendations to generate the final report. Sending in the pre-implementation phase means faxing a paper form; in the post-implementation phase sending means to transfer the form electronically within the HIS to urology.

**Figure 2:**
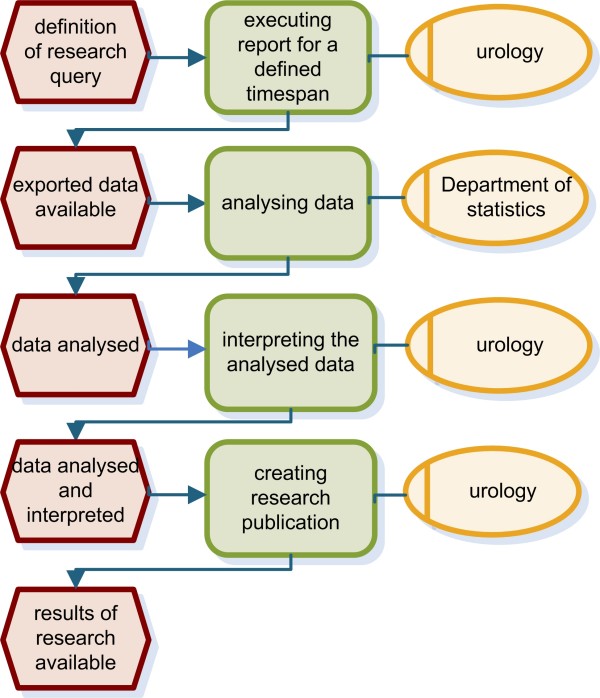
**EPC of the research process**. The research process starts with definition of a research query. Data analysis is done in the department of statistics before urologists interpret the analysed data and create a research publication.

**Figure 3:**
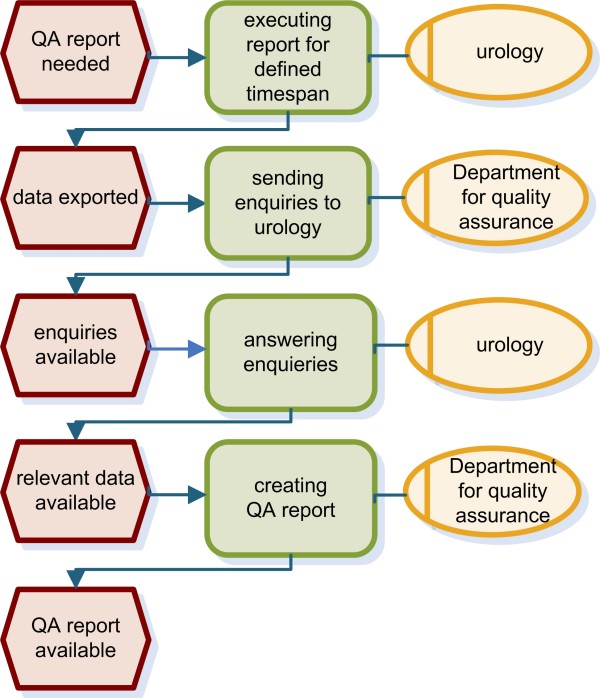
**EPC of the quality assurance process**. For quality assurance QA data is exported and transferred to the department of quality assurance. After answering enquiries by urologists the final QA report is created.

We analysed the process from prostate biopsy to final report. Clinical findings as well as the location of the specimens taken are documented in the department of urology before these data are forwarded to the department of pathology. Pathological findings are transferred to urology again for a final review and annotation. Previously these forms were faxed between the department of urology and the department of pathology and were then sent to the referring physician. Figure 1 presents the workflow using event-driven process chains (EPC) [25,26]. In addition, we analysed communication between the department of urology and the department of statistics for research purposes (Figure 2) as well as processes for quality assurance (Figure 3). Both workflows were also modelled using EPC.

After analysing these processes we implemented two forms of the prostate centre of the university clinic of Münster using the tools of the HIS (ORBIS^® ^from Agfa Healthcare) [27]. Currently ORBIS^® ^is applied to following HIS functions: clinical documentation, administrative documentation, order-entry and scheduling.

**Figure 4:**
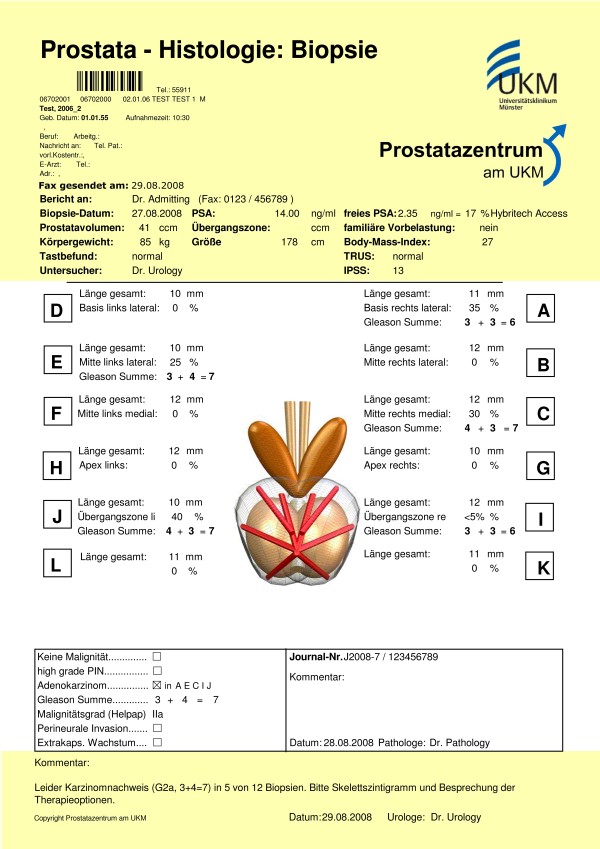
**Prostate biopsy form**. In the upper part fax date ("Fax gesendet am"), the time of biopsy ("Biopsie Datum"), PSA and prostate volume ("Prostatavolumen") are recorded. Up to 12 specimens can be documented with length of the biopsy core ("Länge gesamt"), percentage tumour in biopsy core, and Gleason score ("Gleason Summe"). The yellow part is provided by urologists, the rest is documented by pathologists.

These forms are used for documentation of biopsies and are similar to the previously used paper forms. The main form is shown in figure 4. The urologist (upper yellow part) provides clinical findings like prostate volume, PSA, ultrasound findings and International Prostate Symptom Score (IPSS). Pathological grading systems (Gleason score [28], Helpap-Grading) are filled in by pathologists. After the final assessment of the urologist in the lower part (yellow) of this document it is being sent to the referring physician.

We compared time from biopsy to final report (TBF) between computer-based and paper forms. This time depends on the delivery time of the specimen into pathology but we want to analyse the whole process not considering the delay of the transport of the specimen. There are about six biopsies performed per week so we looked at the data of ten months to get representative figures.

Another indicator is time from biopsy to pathological findings which can be used to monitor delays in this interdisciplinary process. Dates of the first period are manually extracted from the paper-based documentation; dates of computer-based forms are educed by a query using the report generator of ORBIS^® ^[27]. Forms without fax date were excluded.

To assure that data sets from pre- and post implementation phase are comparable, we looked for variables which may influence the time interval between biopsy and final report generation. These are mainly the urologists and the pathologists who participate in the treatment process and the number of patients per week. Therefore we analysed process time for both urologists separately and compared number of patients per week, age and Gleason Score between pre- and post-implementation phase.

To assess clinical acceptance we held contact meetings with clinical users.

To test for significant differences in time span between pre- and post implementation phase we used a non-parametric Mann-Whitney U test. Two sided p-values below 5% were considered significant. The statistical analysis was performed with R [29].

## Results

We created a first report of the paper-based forms (from October 2007 to March 2008) and a second report of the computer-based forms (from April 2008 to July 2008).

Prostate biopsies were mainly performed by two urologists. Table 1 shows that the number of biopsies per physician doesn't change with introduction of HIS forms. The number of patients per week is almost the same in the pre- and post-implementation period, too (5.7 versus 6 per week). Further variables like age of patients (p = 0.51) and the Gleason Score (p = 0.13) have no influence on TBF.

**Table 1:** Basic data of the analysed forms

	*Oct-Mar*	*Apr-Jul*
analysed forms	87	86
urologist A	47 (54%)^1,2^	47 (54.7%)^1^
urologist B	35 (40.2%)^1,2^	39 (45.3%)^1^
Patients/week	5.7	6
Gleason Score	7 (7 to 9)^3^	7 (6 to 7)^3^
Age	67 (59 to 71)^3^	65 (58 to 70)^3^

^1^biopsies per urologist (percentage) ^2^missing percent other physicians ^3^median (IQR)

Number of analysed forms and comparison of the periods concerning physician, age and Gleason Score.

**Figure 5:**
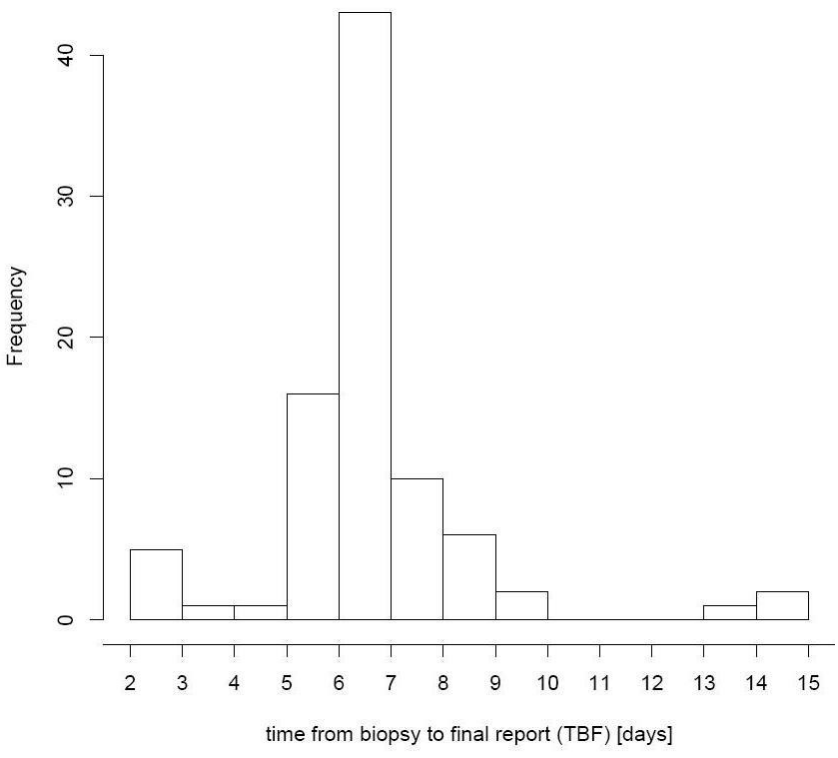
**Pre-implementation phase: Frequency distribution of time from biopsy to final report**. Without the forms in ORBIS^® ^it takes about 1 week after the biopsy to send a final report.

**Figure 6:**
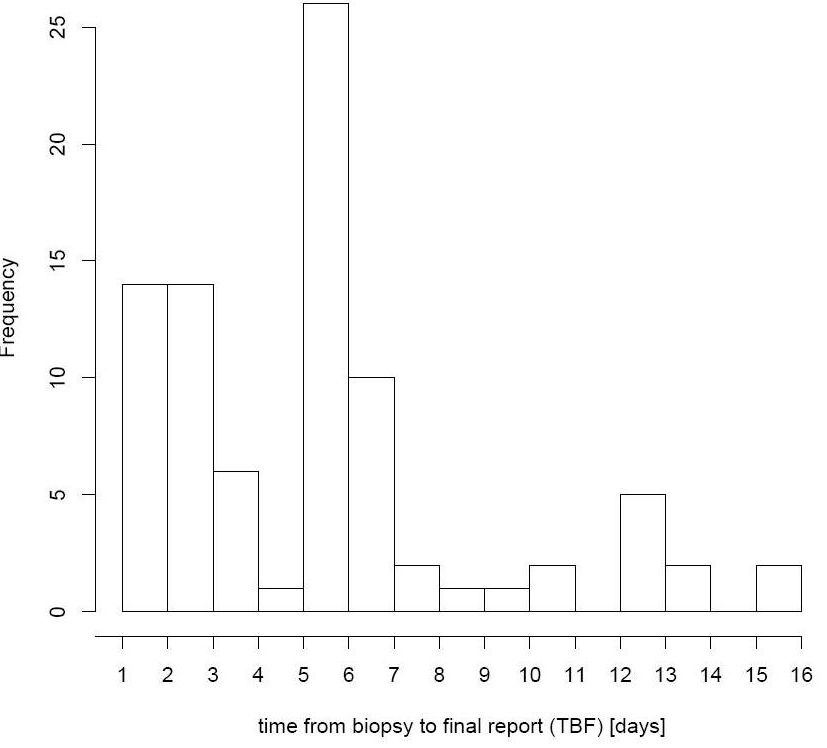
**Post-implementation phase: Frequency distribution of TBF**. With the new forms in ORBIS^® ^it takes about 5–6 days to send a final report. In addition, the proportion of cases with 1–4 days is increased.

Since introduction of computer-based forms, time span from biopsy date until sending of the final report decreased on average more than one day. Figure 5 shows the frequency distribution of the pre-implementation phase while figure 6 shows the frequency distribution of the post-implementation phase.

**Figure 7:**
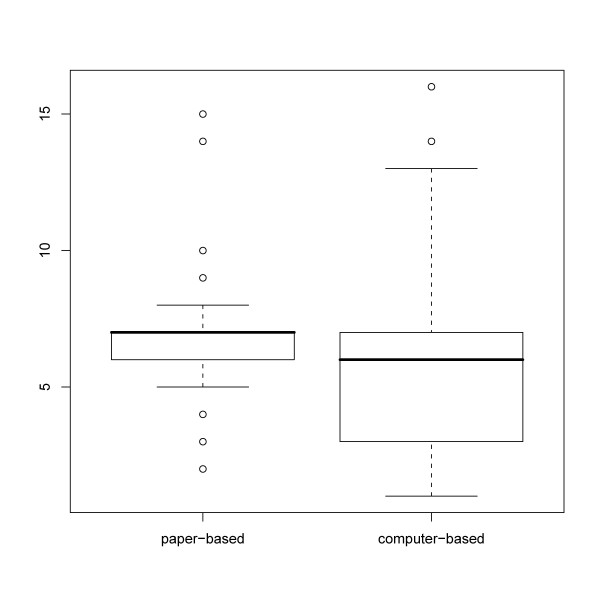
**Boxplots of time from biopsy to final report**. TBF is lower in the computer-based period.

The following boxplots (Figure 7) show that the median of TBF decreases. There are some outliers which can be traced back to delays caused by holidays in both data sets.

The median TBF in the pre-implementation-phase is 7 days versus 6 days in the post-implementation-phase. According to Mann-Whitney-U-test this difference is highly significant with p < 0.0001. Table 2 shows that these time differences are consistent for urologist A and B.

**Table 2:** Descriptive statistics of TBF

	*median (IQR)*^1^	*mean*^1^	*standard deviation*^1^
paper-based	7 (6 to 7)	7.08	4.14
- urologist A	7 (6 to 7.5)	7.06	2.31
- urologist B	7 (7 to 8)	7.13	1.83
computer-based	6 (3 to 7)	5.84	3.56
- urologist A	6 (3 to 7)	6.05	3.13
- urologist B	6 (2 to 7)	5.67	3.96

^1^TBF measured in days

The difference between urologist A and B is not significant both in the paper-based (p = 0.59) and the computer-based (p = 0.25) setting.

From a quality management perspective, the biopsy tissue must be placed in some dilution for one night, so the optimal result is a time span of one day. The average time span was one week. Therefore an average decrease by one day is a relevant result. In contrast to the paper-based system, the electronic system provides a method to regularly measure TBF.

### Secondary use of prostate biopsy data

**Figure 8:**
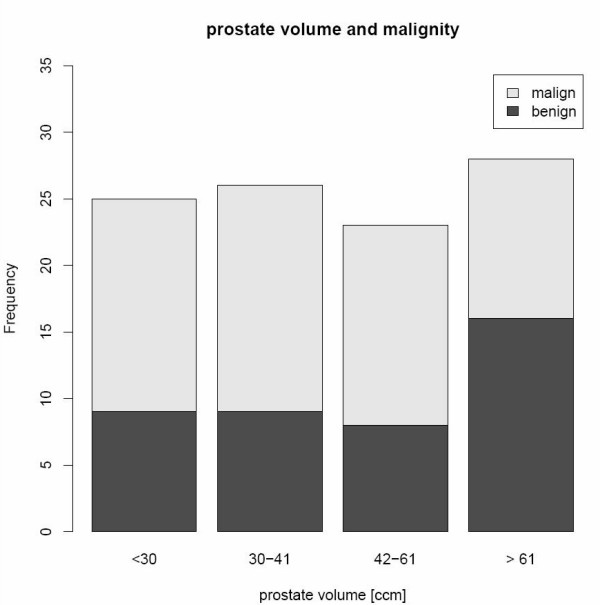
**Prostate volume and malignity**. This report allows assessing associations between the prostate volume and the result of the biopsy (malign or benign).

Furthermore, the electronic system enables to monitor the documentation process regarding completeness, delays and observed coherences. Completeness of documentation is important for data analysis in clinical research. Figure 8 shows the relation of the prostate volume to the result of the biopsy. This figure is based on routine data.

All participating physicians rated decreased time and the possibility to create reports on routine data as relevant advantages of the system. Referring physicians gave positive feedback about fast and well readable final reports in the post-implementation phase.

## Discussion

Our analysis shows that the prostate biopsy workflow can be accelerated significantly by replacing paper-based documentation with electronic HIS forms. The acceptance of this solution was very good because the paper-based forms were nearly identically transformed to HIS and the physicians were already familiar with the HIS.

Advantages like well-readable documents and input support by catalogues and text modules confirm the opinion of the users that such a system is superior to paper-based documentation. Our study is focused on a concrete order-entry process for prostate tumour, but our approach could be extended to optimize the documentation workflow of other tumour entities since many processes are not integrated in the current IT environment of the university hospital.

Furthermore the data are now stored in the HIS. This allows the design of reports to monitor completeness of forms and individual variables like Gleason score. Within the evaluation we observed that the completeness of the routine data was very high partly due to initialisation of some data fields and due to automatic calculations (e.g. exact psa quotient). Especially the well known problem of missing values is eliminated through controlled value lists. Coherences like the relation between prostate volume and malignity are only meaningful if the data are complete. So we observed similar results to Bürkle et al. [30] that increasing computerized documentation can result in better data quality.

Positive effects of computerized documentation could also be found in many studies concerning CPOE. There are "many different commercial and home-made systems available and each of them needs to be carefully evaluated" [14]. But present quantitative studies are few in number and analyse mainly the number of pathological orders [23] or prescriptions [17] as well as effects on patient safety [14,17,21,24]. In most studies effectiveness of a CPOE system is analysed by measuring TAT according to the definition of laboratories as the time from receipt of the specimen until availability of test result [11].

Our main goal was a quantitative analysis of our CPOE system within the HIS by measuring time from biopsy to final report (TBF) to assess duration of the whole medical process. Hurlen et al. introduced a similar indicator by defining report turnaround time (RTAT) to measure not only the technology but take care that the direct "involvement of key actors and [...] are important [...] for sustaining positive results" [31].

In particular, the available literature regarding quantitative effects of CPOE on speed of medical processes is limited. Furthermore most studies analyse separate CPOE systems since many systems aren't integrated in HIS [12] although benefits often realised by institutions using home grown systems [32].

Routine HIS data can be used for quality management, for instance to benchmark time from biopsy to clinical report. It can also be applied for clinical research, for example to analyse the proportion of different subtypes of prostate cancer. For this reason, our study is a "single-source"-approach [33], i.e. to use HIS data for clinical routine as well as research. Especially at a university hospital this is a relevant factor for user acceptance.

There was no introduction phase for the electronic system, because the second phase started directly after the first one. In other studies there were two post-implementations periods [6,7] to eliminate distracting influence of the introduction phase. Because our users were already familiar with the HIS, we expect this effect to be small, but we plan to repeat our analysis in a few months.

In the future we intend to expand and analyse our approach for other departments with tumour documentation.

## Conclusion

Electronic documentation within the HIS can significantly reduce the time from biopsy to final report. From our experience it is important to foster the approach to integrate paper forms into the HIS to gain advantages like time saving, optimized workflow and structured documentation within the HIS. The single source idea is highly accepted by physicians and provides new possibilities like quality monitoring through reports.

## Abbreviations

CDS: clinical decision support; CPOE: computerized physician order entry; EHR: electronic health record; EPC: event-driven process chain; HIS: hospital information system; IPSS: international prostate symptom score; IQR: interquartile range; PSA: prostate specific antigen; QA: quality assurance; RTAT: report turnaround time; TAT: turnaround time; TBF: time from biopsy to final report;

## Competing interests

The authors declare that they have no competing interests.

## Authors' contributions

BB designed the HIS forms and the HIS reports and wrote the manuscript. AS provided the data and contributed clinical requirements. MD reviewed the manuscript. All authors read and approved the final manuscript.

## Pre-publication history

The pre-publication history for this paper can be accessed here:

http://www.biomedcentral.com/1472-6947/9/5/prepub
